# Nitrate reductase ^15^N discrimination in *Arabidopsis thaliana*, *Zea mays*, *Aspergillus niger*, *Pichea angusta*, and *Escherichia coli*

**DOI:** 10.3389/fpls.2014.00317

**Published:** 2014-07-02

**Authors:** Eli Carlisle, Chris Yarnes, Michael D. Toney, Arnold J. Bloom

**Affiliations:** ^1^Department of Plant Sciences, University of CaliforniaDavis, CA, USA; ^2^Department of Chemistry, University of CaliforniaDavis, CA, USA

**Keywords:** nitrate reductase, ^15^N discrimination, natural abundance, enriched ^15^N, plant, fungi, bacteria, kinetic isotope effect

## Abstract

Stable ^15^N isotopes have been used to examine movement of nitrogen (N) through various pools of the global N cycle. A central reaction in the cycle involves the reduction of nitrate (NO^−^_3_) to nitrite (NO^−^_2_) catalyzed by nitrate reductase (NR). Discrimination against ^15^N by NR is a major determinant of isotopic differences among N pools. Here, we measured *in vitro* ^15^N discrimination by several NRs purified from plants, fungi, and a bacterium to determine the intrinsic ^15^N discrimination by the enzyme and to evaluate the validity of measurements made using ^15^N-enriched NO^−^_3_. Observed NR isotope discrimination ranged from 22 to 32‰ (kinetic isotope effects of 1.022–1.032) among the different isozymes at natural abundance ^15^N (0.37%). As the fractional ^15^N content of substrate NO^−^_3_ increased from natural abundance, the product ^15^N fraction deviated significantly from that expected based on substrate enrichment and ^15^N discrimination measured at natural abundance. Additionally, isotopic discrimination by denitrifying bacteria used to reduce NO^−^_3_ and NO^−^_2_ in some protocols became a greater source of error as ^15^N enrichment increased. We briefly discuss potential causes of the experimental artifacts with enriched ^15^N and recommend against the use of highly enriched ^15^N tracers to study N discrimination in plants or soils.

## Introduction

Nitrogen (N) is a key limiting resource in many ecosystems, and so N biogeochemical cycles—particularly the processes of organismal N uptake and assimilation—are of great importance (Epstein and Bloom, 2005). The main N source for most plants is soil inorganic N in the forms of nitrate (NO^−^_3_) and ammonium (NH^+^_4_) ions dissolved in the soil solution. A primary pathway through which inorganic N is converted into organic N is NO^−^_3_ assimilation via the biochemical pathway involving the sequential reactions catalyzed by the enzymes nitrate reductase (NR), nitrite reductase (NiR), glutamine synthetase (GS), and glutamate synthase (GOGAT). Reduction of NO^−^_3_ to NO^−^_2_ via NR is the rate limiting step in this pathway (Robinson et al., 1998; Evans, 2001), and thus the behavior of NR is central to biogeochemical plant-soil interactions as well as to internal plant N cycling (Tcherkez and Farquhar, 2006).

Eukaryotic NRs are members of the sulfite oxidase family of molybdoenzymes. They are found in three forms that use different reductants: an NADH-specific form typically found in higher plants and algae, an NAD(P)H-bispecific form found in higher plants, algae, and fungi, and an NADPH-specific form found only in fungi. All three forms are similar in overall composition (Campbell, 1999; Fischer et al., 2005) and require multimerization for full function, typically as either homodimers or homotetramers (Campbell, 1999; Barbier and Campbell, 2005). Each subunit contains one each of three cofactors: a flavin adenine dinucelotide (FAD), a heme-Fe, and a Mo-molybdopterin (Mo-MPT) cofactor (Campbell, 1999).

Prokaryotic NRs are unrelated to the eukaryotic NRs. They are members of the DMSO (dimethylsulfoxide) reductase family of molybdoenzymes and have an assimilatory (Nas) and two dissimilatory forms. The dissimilatory forms include a membrane-bound respiratory form (Nar) and a periplasmic form (Nap) (Moreno-Vivián et al., 1999). All prokaryotic forms, like all eukaryotic forms, have Mo as a key component of the cofactor that reduces NO^−^_3_. In prokaryotic NRs, however, the Mo is bound to a bis-molybdopterin guanine dinucleotide (MGD) rather than the MPT found in eukaryotes (Berks et al., 1995). The MGD molecule differs from the MPT molecule by the linkage of a guanine monophosphate (GMP) to the phosphate group of MPT (Temple and Rajagopalan, 2000). All prokaryotic NRs are heterotrimeric: they are composed of a Mo-MGD cofactor, a iron-sulfur (FeS) center, and either (*a*) FAD, (*b*) cytochrome b prosthetic, or (*c*) cytochrome c group in the Nas, Nap, or Nar forms, respectively (Moreno-Vivián et al., 1999). The prokaryotic NRs use a variety of electron donors, including ferrodoxin, flavodoxin, and NADH for the Nas enzyme, quinones for the Nap enzymes, and quinols for the Nar enzymes.

Stable N isotopes (^15^N and ^14^N) are a standard method for examining N cycling and transformations on many scales, ranging from global biogeochemical N cycles (Shearer and Kohl, 1986; Schlesinger, 1997; Amundson et al., 2003) to mechanistic enzyme studies (Cleland, 2005). Two approaches are common, natural abundance and ^15^N-enriched. In the first approach, one monitors how the natural abundance ^15^N/^14^N ratio of various compounds in a pathway differs from a reference, typically that of atmospheric N_2_ gas (0.37% ^15^N). An advantage of the natural abundance approach is that no additional N need be added that might perturb the experimental system. For example, inorganic N (e.g., NO^−^_3_), if added above background levels, can inhibit N_2_-fixation or alter N transformations and assimilation (Sprent, 1979).

In the second approach, one employs a ^15^N-enriched reactant as a tracer and monitors δ^15^N differences through various N pools (Hauck and Bremner, 1976; Androsoff et al., 1995). Slight differences in substrate ^15^N content and variability introduced through mixing and fractionation of different N pools in the soil and the plant are overwhelmed by the tracer signal. This approach is not typically used to examine enzymatic isotope fractionation. Regardless of the experimental approach, a key assumption is that ^15^N behaves identically to ^14^N in biological systems except for the differences due to mass (Hauck and Bremner, 1976).

When environmental or substrate concentrations of NO^−^_3_ are limiting, little to no discrimination against the ^15^N isotope is observed during NO^−^_3_ assimilation (Kolb and Evans, 2003). Discrimination against the ^15^N isotope has been demonstrated to occur only when the NO^−^_3_ concentration exceeds plant needs or uptake ability. The key to understanding these observations is that isotopic discrimination is only detected to the extent that both isotopic species are equilibrated between the plant and the environment during the course of the measurement (Kolb and Evans, 2003). In such cases, plant NR has typically been cited as discriminating against ^15^N by 15‰ when the enzyme is supplied adequate amounts of NO^−^_3_ substrate containing N isotopes at natural abundance (i.e., product NO^−^_2_ is depleted in ^15^N relative to substrate NO^−^_3_ by 15 parts per 1000; Mariotti et al., 1982; Ledgard et al., 1985; Evans et al., 1996; Tcherkez and Farquhar, 2006). Nonetheless, reported values for NR ^15^N discrimination vary from 5 to 30‰ (Schmidt and Medina, 1991; Granger et al., 2004; Needoba et al., 2004; Tcherkez and Farquhar, 2006).

Previously, we obtained significantly different results for the rate of NO^−^_3_ assimilation in plant shoots when using ^14^N or ^15^N as a tracer (Bloom et al., 2010). *Arabidopsis*, grown under ambient CO_2_ concentrations with natural abundance NO^−^_3_ nutrition and pulsed with 25% enriched ^15^NO^−^_3_, demonstrated 20–30% less NO^−^_3_ assimilation than *Arabidopsis* grown on 99% enriched ^15^NO^−^_3_ and pulsed with natural abundance NO^−^_3_. In wheat, these experimental protocols resulted in similar trends (Bloom et al., 2010). These differences in assimilation rates may have been confounded by artifacts of the isotope measurements by the isotope ratio mass spectrometer (IRMS).

In this study, we examined how NR discrimination varies among different organisms, and determined whether ^15^N enrichment of substrate NO^−^_3_ affects the measurement of NR discrimination, which may have caused the unusual results in our previous work (Bloom et al., 2010). We conducted *in vitro* experiments on five purified NRs from plants, fungi, and a bacterium to address two hypotheses: (1) natural abundance ^15^N discrimination is similar among NRs; (2) substrate NO^−^_3_^15^N-enrichment affects instrumental analysis of samples and the estimation of ^15^N discrimination.

## Materials and methods

We measured ^15^N-isotope discrimination by commercial preparations of five different nitrate reductases. Four of these enzymes are assimilatory nitrate reductases including that from *Arabidopsis thaliana* (AtNaR2 NADH:nitrate reductase E.C. 1.7.1.1), *Aspergillus niger* (NAD(P)H:nitrate reductase E.C. 1.7.1.2), *Pichia angusta* (YNaR1 NAD(P)H:nitrate reductase E.C. 1.7.1.2), and *Zea mays* (maize; NADH:nitrate reductase E.C. 1.7.1.1); these were assessed using similar protocols. The fifth is a prokaryotic NR (*Escherichia coli*, cytochrome:nitrate reductase E.C. 1.9.6.1), which is a dissimilatory reductase (Nar form); this enzyme required a different experimental protocol. The maize, *Aspergillus*, and *E. coli* enzymes are isolated from the native organisms, while the *Arabidopsis* and yeast enzymes are recombinant enzymes expressed in the yeast *Pichia pastoris*. The *Aspergillus* and *E. coli* enzymes were purchased from Sigma, and the rest were purchased from NECi (nitrate.com; also see Barbier et al., 2004). We were unable to detect any nitrate or nitrite in the enzyme preparations (Miranda et al., 2001).

### Assimilatory nitrate reductases

One unit of enzyme activity is defined as the amount of NR that is capable of catalyzing the conversion of 1 μmol nitrate to nitrite per minute at optimal pH and temperature. Assimilatory nitrate reductase analyses were carried out by reconstituting the freeze-dried *Arabidopsis*, yeast, and maize enzymes in 25 mM potassium phosphate, 0.1 mM Na_2_EDTA, pH 7.5, at 22–25°C, to a final concentration of 1 unit/mL. For the *Aspergillus* enzyme, a 100 mM potassium phosphate buffer, 0.1 mM Na_2_EDTA, pH 7.5 was used. The NR *K*_m_ for NO^−^_3_ in *Arabidopsis* and maize ranges from 15 to 40 μM (Su et al., 1997; Campbell, 1999), 199 μM for *Aspergillus* (Gilliam et al., 1993), and 470 μM for the dissimilatory NR in *E. coli* (Adams and Mortenson, 1982). There is no information for the specific yeast NR used in our study, but NRs from other genera of yeast have a *K*_m_ ranging from 110 (Hipkin et al., 1986) to 740 μM (Morozkina et al., 2005).

The enzyme reaction solution contained 1 unit (1 mL) of reconstituted NR, 165 mL of additional phosphate buffer (pH 7.5), and 70 μmol of NADH or NADPH for the plant and fungal enzymes, respectively. Additionally, the *Aspergillus* solution contained 4 μmol of FAD. After mixing, 10 mL of solution was added to each of 16 sterile 15 mL centrifuge tubes. One mL of KNO_3_ solution (0.2 M KNO_3_) was then added to each centrifuge tube, and the tubes were placed into a water bath at 30°C (25°C for *Aspergillus*) for 30 min. The use of 0.2 mmol of KNO_3_ in the reaction tubes was calculated to provide a surplus of NO^−^_3_ for the enzymatic reaction, while minimizing the possibility that residual NO^−^_3_ might saturate the mass spectrometer detectors. Therefore, the enzyme reactions were not necessarily operating under NO^−^_3_ saturating conditions. The KNO_3_ was enriched with different levels of ^15^N from natural abundance to >99 atom % ^15^N to examine how substrate ^15^N enrichment influences the estimation of NR ^15^N discrimination using an IRMS. At the end of the incubation, samples were placed into 20 mL scintillation vials. The reaction was halted by adding 0.25 mL of 1 M HCl and swirling the vials (decreasing pH to below 4). After 1 min, 40 μL of 10 M KOH were added to the solution, raising its pH to 10–12 to prevent oxidation of NO^−^_2_ to NO^−^_3_. Concentrations of NO^−^_2_ and NO^−^_3_ were determined using the Griess reaction (Miranda et al., 2001).

### Dissimilatory nitrate reductase

The reaction catalyzed by dissimilatory *E. coli* NR requires reducing conditions. The enzyme was reconstituted in 2 mL of cold degassed deionized water and added to 55 mL of degassed buffer (0.125 M MOPS, pH 7.0) containing 30 mg benzyl viologen (BV; 1,1′-Dibenzyl-4,4′-bipyridinium dichloride, Sigma) as an electron donor. Ten milliliter of buffer/enzyme solution were added to 15 mL centrifuge tubes containing 0.2 mmoles of KNO_3_ of various ^15^N enrichments and 0.25 mL of a sodium dithionite/sodium bicarbonate solution (500 mg of each added to 50 mL degassed DI H_2_O) to maintain reducing conditions. If the blue color of the reduced BV dye started to fade, additional dithionite solution was added to the vial. The vials were incubated in a water bath at 30°C for 90 min. To stop the reaction, vials were shaken vigorously to oxidize the BV. At the end of the incubation, 75 μl of 10 N NaOH was added to each tube to minimize conversion of NO^−^_2_ to NO^−^_3_.

### Preparation of bacterial cultures for headspace N_2_O analysis

For all forms of the NR enzyme, we analyzed the ^15^N isotope composition of the headspace N_2_O produced through bacterial denitrification of NO^−^_3_ and NO^−^_2_. Bacterial cultures and solutions were prepared as in Böhlke et al. (2007). The bacterial cultures used in this study were *Stenotrophomonas nitritireducens* (ATCC# BAA-12) and *Pseudomonas chlororaphis* (ATCC# 43928), both of which were obtained from the American Type Culture Collection (www.atcc.org). Both species lack the enzymatic ability to reduce N_2_O to N_2_. *S. nitritireducens* is limited to reducing NO^−^_2_, while *P. chlororaphis* is capable of reducing both NO^−^_3_ and NO^−^_2_ to N_2_O. This specificity allowed a sequential reduction of our experimental samples to estimate the ^15^N concentrations of the NO^−^_3_ and NO^−^_2_ independently.

The denitrifying bacteria were streaked on tryptic soy agar (TSA) plates composed of TSA (40 g/L) and tryptic soy broth (TSB; 10 g/L) for *S. nitritireducens*, and TSA (40 g/L) and KNO_3_ (1 g/L) for *P. chlororaphis*. The TSA plates for the *S. nitritireducens* cultures were prepared with 10 mM Na_2_WO_4_ to minimize any potential NO^−^_3_ reduction (Nielsen et al., 2004). Both cultures were grown aerobically at 22–24°C. After 5–7 days, bacterial colonies were streaked onto a second plate. If re-plated more than 2 or 3 times, higher background levels of N_2_O were observed for both bacteria. Therefore, all N_2_O analyses were made using colonies from the second plate; additional analyses required starting the process over from the original frozen aliquot.

Individual *S. nitritireducens* colonies were transferred 4–5 days after streaking to sterile 50 mL centrifuge tubes with 35 mL of a growth medium containing TSB solution (15.0 g/L), (NH_4_)_2_SO_4_ (0.25 g/L), and KH_2_PO_4_ (2.45 g/L). Tubes were capped leaving an air headspace and agitated on a reciprocal shaker for 1–2 days at 22°C. The tubes were opened daily to allow for aerobic growth. The contents of the centrifuge tubes were combined and an antifoaming agent was added, 2–3 drops of Antifoam B (Sigma) per 105 mL solution. Four milliliter of the combined solution were pipetted into each 20 mL vial, which was capped with a septum and crimped, and then placed on a manifold that purged the vials with N_2_ gas for 4 h to remove any residual oxygen, CO_2_, and N_2_O. The contents of these vials were used for ^15^N analysis of NO^−^_2_, as well as for providing substrate for the NO^−^_3_ reducing *P. chlororaphis* cultures.

*Pseudomonas chlororaphis* cultures were used to inoculate a starter tube containing 1.5 mL of TSB solution (30 g/L), KNO_3_ (1 g/L), and (NH_4_)_2_SO_4_ (0.066 g/L), which was placed on a reciprocal shaker for 1 day. TSB solution (400 mL) was added to a 500 mL media bottle, which was inoculated with the contents of the starter tube and sealed tightly. The media bottle was placed on the reciprocal shaker, and the bacteria were allowed to grow for 6–10 days under anaerobic conditions. At the end of the 6–10 days, the solution was centrifuged (10 min at 20°C and 6500 rpm). The supernatant was decanted into a large beaker and the pellets remaining in the centrifuge tubes were resuspended in 60 mL of the supernatant solution. An antifoam agent—one drop of Antifoam B (Sigma) per 60 mL—was added and the concentrated bacterial solution was mixed thoroughly. Two milliliter of this solution were pipetted into each 20 mL headspace vial, capped with a septum and crimped, and then purged for 4 h on the manifold system with N_2_ gas. The manifold system was carefully sterilized between sample sets.

### Bacterial NO^−^_2_ and NO^−^_3_ reduction to N_2_O

Solutions from the enzyme reaction were added to the purged *S. nitritireducens* vials to determine the ^15^N enrichment of N_2_O derived from NO^−^_2_. The amount of solution added to the vial was determined by the concentration of the NO^−^_2_ in the solution. A target value of 0.025 μmol of NO^−^_2_ were added to each vial. This corresponded to volumes ranging from 0.25 to 1.5 mL depending on the enzyme. The vials were then placed on a reciprocal shaker for 24 h. At the end of 24 h, a sample was removed from the vial using a gas-tight syringe and injected into a vial containing *P. chlororaphis*. Again, a volume of solution equivalent to 0.025 μmol of NO^−^_3_ was added to the vials (volumes ranged from 25–200 μL). Amounts of N added to all samples were matched to ensure that the samples remained in the linear range of the detectors in the IRMS. The *P. chlororaphis* vials were placed on the reciprocal shaker.

Once the subsample had been removed from the vials containing *S. nitritireducens*, and after 24 h incubation for the *P. chlororaphis* vials, 0.2 mL of 10 M NaOH were added to both sets of vials killing the bacteria and removing any residual headspace CO_2_. All vials were then taken to the UC Davis Stable Isotope Facility where they were analyzed for [N_2_O] and ^15^N (ThermoFinnigan GasBench + PreCon trace gas concentration system interfaced to a ThermoScientific Delta V Plus IRMS, Bremen, Germany). To control for potential contamination, blanks (including bacterial solution alone and with DDI water), natural abundance NO^−^_2_ and NO^−^_3_ samples, and appropriately ^15^N-enriched NO^−^_2_ and NO^−^_3_ standards, were included in each sample run. The blanks also allowed us to correct for any N_2_O derived from residual NO^−^_3_ remaining in the *P. chlororaphis* solution and instrumental leaks. Samples of the enzyme reaction solution were also tested for inorganic N contamination and found not to contain detectable amounts. No remaining inorganic N was found in the spent nutrient solution after the bacterial incubations. Thus, conversion of the NO^−^_3_ and NO^−^_2_ to N_2_O was complete and quantitative.

### Analysis of ^15^N

We defined ^45^R and ^46^R as the ratios of measured ion peak areas [^45^R = (*m/z* 45)/(*m/z* 44), and ^46^R = (*m/z* 46)/(*m/z* 44)]. ^15^R or the ratio of ^15^N/^14^N of the N_2_O headspace was determined as:

(1) 15R= R45− R172

where ^17^R (^17^O/^16^O) is fixed at the isotopic oxygen signature of ambient N_2_O (^17^R = 0.00038855; Kaiser et al., 2003). ^15^R may equivalently be determined using ^46^R as described in Stevens and Laughlin (1998):

(2) $${}^{15}R={-}^{17}R+\sqrt{\left[\left({}^{17}R\right){+}^{46}R{-}^{18}R\right]}\;$$

where ^18^R (^18^O/^16^O) is fixed at the isotopic oxygen signature of N_2_O (δ^18^O = 44.62 and ^18^R = 0.0020947; Kaiser et al., 2003).

Isotope analyses are often expressed in terms of either δ^15^N or atom % ^15^N. δ^15^N is expressed as the per mil difference between the ^15^N/^14^N ratio of a sample relative to the primary reference material for nitrogen, atmospheric N_2_(“Air,” 0‰):

(3) $$\delta^{15}N(‰)=\;\left(\frac{\frac{{}^{15}N_{sample}}{{}^{14}N_{sample}}}{\frac{{}^{15}N_{standard}}{{}^{14}N_{standard}}}-1\right)*1000$$

For all analyses, initial measurements are made relative to a working reference gas of the same species (i.e., N_2_O, N_2_), co-measured during analysis. Subsequently, all measurements were corrected using working standards previously calibrated to the following standard reference materials IAEA-N1, IAEA-N2, IAEA-N3, USGS-40, and USGS-41.

There are, however, no internationally accepted reference standards for N_2_O at high ^15^N enrichments and, because of this, we compared the ^15^N/^14^N ratio of the sample N_2_O to the ^15^N/^14^N ratio of the substrate NO^−^_3_ as determined by the quantitative combustion of substrate NO^−^_3_ to N_2_ (Stickrod and Marshall, 2000; See Supplemental Table 1) via a PDZ Europa ANCA-GSL Elemental Analyzer interfaced to a PDZ Europa 20-20 IRMS (Sercon Ltd., Cheshire, UK) according to:

(4) $$\delta^{15}N(‰)=\left(\frac{\frac{{}^{15}N_{sample}}{{}^{14}N_{sample}}}{\frac{{}^{15}N_{substrate}}{{}^{14}N_{substrate}}}-1\right)*1000$$

This approach minimizes the impact of absolute errors in calibration because only differences between the substrate and product are considered, and the δ notation is very sensitive to large absolute differences between the sample and reference ratios (Fry, 2006). Also, in order to examine the performance of the bacterial reduction method at high levels of ^15^N enrichment, we calculated the atom % ^15^N from the atomic ratios, ^15^R:

(5) $$Atom{\%}^{15}N=\frac{\left(100{*}^{15}R\right)}{\left(1{+}^{15}R\right)}$$

Alternatively, the atom % ^15^N could be determined from the molecular ratios (e.g., ^45^R and ^46^R) as outlined in Stevens and Laughlin (1998).

The kinetic isotope effect (KIE) for a chemical reaction is the relative reactivity of molecules labeled with different isotopes (Farquhar et al., 1989). Data for competitive KIE experiments is generally taken at different values of fractional reaction of the initial substrate and fitted to Equation (6a), where *f* is fractional conversion of the initial substrate, *R*_0_ is the isotopic ratio of the initial substrate (equal to the isotopic ratio of the product at 100% conversion), and *R_f_* is the isotopic ratio of the product at fractional reaction *f*. Here, because all reactions were halted at very low conversions of the initial NO^−^_3_ (0.5% maximal conversion), the KIE is well approximated by the molar ^15^N/^14^N ratio of the initial nitrate substrate to the nitrite product (Equation (6b); Bigeleisen and Wolfsberg, 1958):

(6a) $$KIE=\frac{\text{ln}(1-f)}{\text{ln}\left(1-f\times (R_{f}/R_{0})\right)}\;$$

(6b) $$KIE=\frac{R_{0}}{R_{f}}$$

Therefore, the KIE is related to the measured isotopic discrimination (Δ) by

(7) Δ=KIE−1

Alternatively, it can be derived by the following equation (Evans, 2001):

(8) $$\Delta =1000\left[\frac{\delta^{15}N_{0}-\delta^{15}N_{f}}{1+\frac{\delta^{15}N_{f}}{1000}}\right]$$

where δ^15^*N*_0_ and δ^15^*N_f_* are the ^15^N content of the initial substrate and the product at fractional reaction *f*, respectively. Enzyme discrimination data will be presented using the Δ notation for the remainder of this paper.

The bacterial reduction method was compared with a chemical reduction method to examine potential bacterial discrimination against ^15^N during the reduction of NO^−^_2_ to N_2_O. For the chemical reduction, we used sulfamic acid to reduce NO^−^_2_ to N_2_O (Granger and Sigman, 2009). As a result of this reaction, the solution NO^−^_2_ supplies one of the N atoms in the N_2_O molecule, while sulfamic acid supplies the other (Granger and Sigman, 2009). No remaining NO^−^_3_ was observed in the samples after the reduction.

### Statistical analysis

The experiments were formulated as a randomized complete block design. Blocks were represented by the enzyme batch to control for inter-batch variation in enzyme activity and purity. Depending on the particular enzyme, a single batch of enzyme was used to produce from one to three repetitions of each ^15^NO_3_ concentration to NO^−^_2_. A linear mixed effects model (PROC MIXED, SAS 9.1, Cary, NC, USA) was used to analyze the data.

## Results

NR discrimination at natural abundance ranged (mean ± *SE*) from 22.5 ± 0.8‰ for *Arabidopsis* to 31.6 ± 2.3‰ for *E. coli* (Table 1). Interestingly, the two recombinant NRs (*Arabidopsis* and yeast) had the lowest discrimination among the enzymes. The average discrimination for the non-recombinant assimilatory eukaryotic NRs was 29.4 ± 0.4‰, significantly greater (*p* < 0.0001) than the average of 22.8 ± 0.5‰ for the recombinant NRs. The isotope discriminations of the maize and *E. coli* enzymes were significantly greater than that of the enzymes from *Arabidopsis* (*p* = 0.0171 and *p* = 0.0052, respectively) and yeast (*p* = 0.0254 and *p* = 0.0052, respectively). The KIEs had similar values among all species (Table 2) at natural abundance ^15^N.

**Table 1:** **Observed ^15^N natural abundance isotope discrimination (Δ in ‰, *n* = 6–10) for the five tested NRs**.

**^15^N enrichment**	***Arabidopsis***	**Yeast**	***Z. mays***	***A. niger***	***E. coli***
0.37%	22.5	23.0	30.9	28.1	31.6
	(0.8)	(0.5)	(1.5)	(0.4)	(2.3)

*Standard errors are presented in parentheses*.

**Table 2:** **Kinetic isotope effects (KIEs) for the five tested NRs**.

**^15^N enrichment**	***Arabidopsis***	**Yeast**	***Z. mays***	***A. niger***	***E. coli***
0.37%	1.023	1.023	1.031	1.028	1.032
	(0.001)	(0.002)	(0.001)	(0.003)	(0.003)
0.5%	1.029				
	(0.002)				
1%	1.034				
	(0.001)				
10%	1.052	1.051	1.056		
	(0.001)	(0.001)	(0.001)		
25%	1.053	1.053	1.058		
	(0.002)	(0.002)	(0.001)		
50%	1.075	1.068	1.071	1.078	1.078
	(0.002)	(0.001)	(0.002)	(0.004)	(0.002)
75%	1.278	1.179	1.186		
	(0.018)	(0.010)	(0.020)		
99%	4.483	3.470	2.723	3.957	3.482
	(0.317)	(0.536)	(0.026)	(0.450)	(0.345)

*Standard errors are presented in parentheses. With highly ^15^N enriched substrate pools, it is more appropriate to assess isotope discrimination as the KIE. Increasing ^15^N enrichment results in larger deviation from natural abundance KIEs and greater variation in the measurements*.

**Figure 1:**
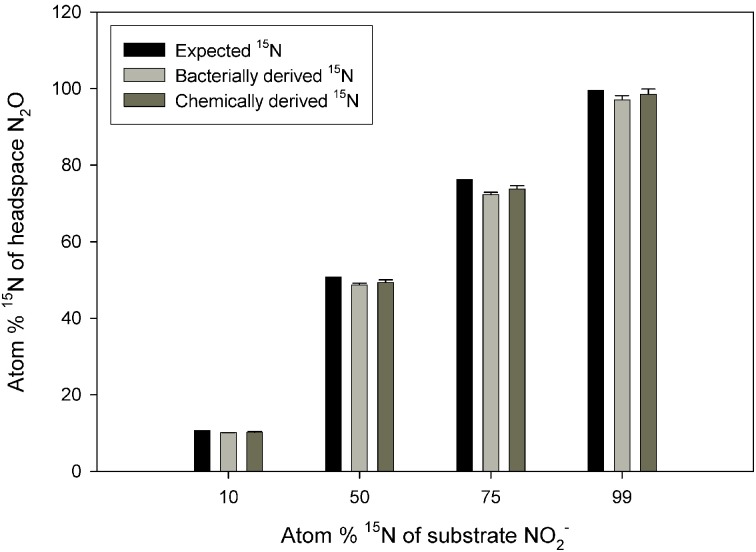
**Observed ^15^N concentration in N_2_O across a range of substrate ^15^N enrichments**. The N_2_O was produced from reduction of NO^−^_2_ to N_2_O via *S. nitritireducens* cultures (bacterially derived) or via sulfamic acid (chemically derived). Also plotted are the expected ^15^N concentrations. The bacterial and chemical data were calculated using Equation (5), and both approaches used the same NO^−^_2_ substrate. The expected plot assumes a constant ^15^N discrimination of −29.44‰. Error bars represent the standard errors of the means (*n* = 5).

There was no significant difference between the values obtained via the chemical and bacterial reduction methods (Figure 1; *p* = 0.5633), although the data from the chemical reduction method tended to be closer to the expected ^15^N enrichment at high ^15^N enrichments.

**Figure 2:**
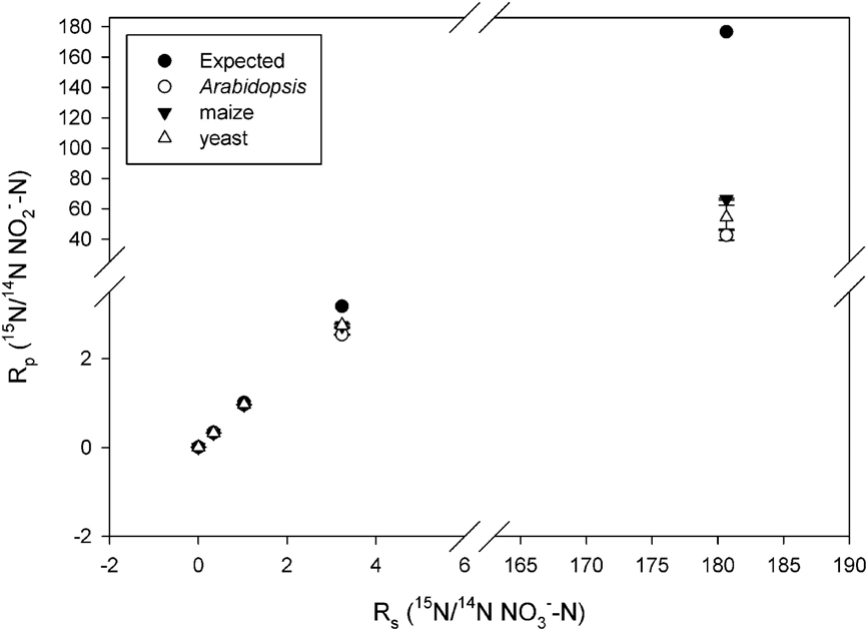
**Plot of R_p_ (ratio of ^15^N/^14^N of the product NO^−^_2_) vs. R_s_ (ratio of the ^15^N/^14^N of the substrate NO^−^_3_) produced by the listed nitrate reductases (NR) when measured at natural abundance, 25, 50, 75, and 99 atom % ^15^N**. Error bars represent the standard error of the means (*n* = 5–9). The expected plot was produced assuming that the NR KIEs (i.e., discrimination) were constant at 1.030.

Observed KIEs (*R*_0_/*R_f_*) increased with increasing substrate ^15^N enrichment for all enzymes (*p* < 0.001; Figure 2, Table 2), particularly above 50 atom % ^15^N. The *Arabidopsis* enzyme showed the greatest deviation from expected, but all enzymes produced significantly less ^15^N-NO^−^_2_ than expected at ^15^N enrichments above 50 atom % (*p* < 0.001).

## Discussion

### Natural abundance

The results show that ^15^N discrimination in the NR-catalyzed reaction is similar across eukaryotic NRs, particularly among non-recombinant NRs. All NRs had ^15^N discrimination falling between 22 and 32‰ (corresponding to KIEs of 1.023 and 1.033, respectively; Table 2). This is higher than the 15‰ typically cited in the literature (e.g., Robinson, 2001; Werner and Schmidt, 2002). The relatively high ^15^N discrimination observed here may partially derive from differences in how the measurements were made. Other work has generally focused on *in vivo* discrimination (e.g., Mariotti et al., 1982) or *in vitro* experiments using isolated organelles or cytosolic fractions (e.g., Ledgard et al., 1985). *In vitro* experiments with purified enzymes typically produce greater isotopic discrimination than experiments with cellular organelles, multiple enzymes, or whole organisms because additional factors diminish ^15^N discrimination as the catalytic system becomes more complex (Werner and Schmidt, 2002). We performed *in vitro* assays using purified NRs and electron donors, providing unlimited substrate, and operating under optimal conditions to prevent subsequent conversion of the product NO^−^_2_. Olleros-Izard (1983: cited in Schmidt and Medina, 1991) used a similar approach with purified corn NR and observed a discrimination of 30‰, which agrees with our values for non-recombinant enzymes. Karsh et al. (2012) using purified enzymes and the bacterial reduction method found that the assimilatory NR from *A. niger* discriminated against ^15^NO^−^_3_ by 26.6 ± 0.2‰ (Karsh et al., 2012), again a value similar to ours.

The four assimilatory eukaryotic NRs (*Arabidopsis*, yeast, maize, and *A. niger*) are structurally similar, although the two recombinant enzymes (*Arabidopsis* and yeast) lack a portion of the complete native enzyme. For example, the recombinant *Arabidopsis* NR, which is natively homotetrameric, is missing in three of its four subunits the Mo-MPT cofactor, and thus the NO^−^_3_ reducing site (Skipper et al., 2001). Such differences in structure may account for the lower ^15^N discrimination observed in the recombinant NRs than in the maize and *A. niger* enzymes (Table 1). Some of the observed difference between the *Arabidopsis* and maize NRs may be due to differences between monocot and eudicot NRs; however, there is no published evidence for this possibility. Differences in glycosylation between native enzymes and recombinant enzymes expressed in *P. pastoris* also might affect enzymatic activity and possibly isotope discrimination (e.g., Henriksson et al., 2003).

Despite the structural disparity (Moreno-Vivián et al., 1999) between the eukaryotic and prokaryotic NRs, the *E. coli* NR did not grossly differ in NR ^15^N discrimination from the other NRs tested.

The electron donor did not strongly affect ^15^N discrimination. The plant-derived NRs (*Arabidopsis* and maize) used NADH as an electron donor, while the fungal NRs used NADPH. The similar level of discrimination that we found despite the difference donors supports the hypothesis that substantial structural differences among the eukaryotic enzymes are largely localized to the FAD binding domain (Campbell, 1999; Karsh et al., 2012). The dissimilatory *E. coli* NR used BV as an artificial electron donor, which provides electrons to the Mo-MGD cofactor directly, in contrast to the biological donor quinol, which provides electrons through the FeS or cytochrome c cofactors. No studies have measured the effects of the biological electron donor on ^15^N discrimination in nitrate reduction in prokaryotes, and it is not clear whether the use of a different electron donor would affect ^15^N discrimination. Nonetheless, use of an artificial electron donor (e.g., methyl viologen) rather than NADH had little to no influence on eukaryotic NR ^15^N discrimination (Karsh et al., 2012).

### Estimation of NR discrimination using enriched ^15^N

The systematic and large deviation from expected values of the KIEs at high ^15^N enrichment (particularly >50 atom %; Figure 2) cannot derive from substrate dependent changes in the rate limiting nature of N-O bond cleavage because the observed KIEs are greater than their theoretical limits (Tcherkez and Farquhar, 2006). Our measurements at high ^15^N enrichment were affected by artifacts. Similar artifacts may have affected the ^15^N pulse labeling experiment in our previous work (Bloom et al., 2010). The following discusses some of these potential artifacts.

First, although the sequential bacterial reduction method to assess ^15^N discrimination at natural abundance ^15^N worked very well, we observed increasingly large standard errors and anomalously low ^15^N enrichment in the N_2_O derived from NO^−^_3_ reduction by *P. chlororaphis* as ^15^N NO^−^_3_ enrichment increased (Supplemental Table 2). At enrichments of 99 atom %, the N_2_O derived from the NO^−^_2_ was substantially more enriched in ^15^N than the N_2_O derived from NO^−^_3_ (97.6 vs. 58.3 atom % ^15^N; Supplemental Table 2). If we used a different NO^−^_3_ reducing bacterium, *Pseudomonas aureofaciens* (Sigman et al., 2001), the observed differences were even larger (97.6 vs. 7.9 atom %; Supplemental Table 2). By contrast, *S. nitritireducens* produced N_2_O from NO^−^_2_ with ^15^N enrichment comparable to that produced via chemical reduction (Figure 1). These results suggest that the sequential bacterial reduction method should be limited to ^15^N enrichments of less than 10 atom %. The reason for the variation among the bacterial strains is not clear, although ages of the bacterial strains or possible bacterial contamination may play a role. NO^−^_3_ concentrations in the spent bacterial reduction solutions contained no measurable NO^−^_3_ after reduction, so the reaction was apparently quantitative (data not shown).

Second, instrument performance and the mathematical models currently available for the calculation of ^15^N in N_2_O at high levels of ^15^N enrichment are likely responsible for some of the observed variation. Using an IRMS rather than using an elemental analyzer coupled with a mass spectrometer contributed to the observed error, particularly at ^15^N enrichments greater than 50%, however, the use of the IRMS was necessary to measure NR discrimination at natural abundance ^15^N concentrations. High enrichments of ^15^N lead to problems with IRMS collector saturation, and we could not measure the 44 peak necessary for the calculation of the mass ratios.

The results from highly ^15^N enriched substrates presented here illustrate a more general methodological issue regarding stable N isotopes: one should avoid the use of highly ^15^N enriched substrates in discrimination studies, particularly if small experimental changes in ^15^N concentration are expected. High ^15^N enrichments exacerbate methodological difficulties in sample preparation, measurement, and instrument calibration. This ultimately increases the final experimental error and unnecessarily confounds experimental outcomes.

Indeed, the problems in the use of highly enriched ^15^N encountered here may affect measurements at larger scales. For example, it is common in agricultural field studies to use enriched ^15^N as a tracer for the cycling of N among different soil pools. Experiments conducted using enriched ^15^N tracers consistently estimate lower N cycling rates than those conducted using natural abundance or non-isotopic methods (e.g., Hauck and Bremner, 1976; Androsoff et al., 1995; Ladha et al., 2005). Other factors beyond the measurement issues described above certainly play a role in the observed differences among these approaches, but artifacts linked to the use of high ^15^N enrichments such as those observed here may contribute to these differences.

In conclusion, despite the use of different (i.e., natural and artificial) electron donors, NR ^15^N discrimination at natural abundance levels of ^15^N varied between 22 to 32‰ among four assimilatory eukaryotic NRs as well as a prokaryotic dissimilatory NR. These discriminations are similar to those observed or reported in other recent studies (Tcherkez and Farquhar, 2006; Karsh et al., 2012). Difficulties encountered using heavily ^15^N-enriched samples demonstrate the undesirability of protocols requiring such labeling for the measurement of enzyme isotope discrimination.

### Conflict of interest statement

The authors declare that the research was conducted in the absence of any commercial or financial relationships that could be construed as a potential conflict of interest.
