# Supplementary material for: Nitrate reductase 15N discrimination in Arabidopsis thaliana, Zea mays, Aspergillus niger, Pichea angusta, and Escherichia coli
Source: Front Plant Sci. 2014 Jul 2;5:317. doi: 10.3389/fpls.2014.00317 (PMC4078254; doi:10.3389/fpls.2014.00317)
Supplement: Supplementary file 1 [file DataSheet1.DOCX]

Supporting information

Supplement 1: Means and standard errors (in parentheses) of the δ^15^N, measured N, expected N, and % recovery of natural abundance KNO_3_ combusted to N_2_ using a PDZ Europa ANCA-GSL Elemental Analyzer interfaced to a PDZ Europa 20-20 IRMS (Sercon Ltd., Cheshire, UK). Sample masses ranged from 10 to 150 μg. Samples combusted for the measurement of our experimental ^15^N enriched KNO_3_ were at least 80 ug N.

|  | δ^15^N (‰) | Measured N (ug) | Expected N (ug) | Recovery (%) |
| --- | --- | --- | --- | --- |
| 10 μg N | -4.17 (0.38) | 9.59 (0.30) | 10.12 (0.25) | 94.76 (0.87) |
| 20 μg N | -3.77 (0.21) | 19.28 (0.18) | 20.48 (0.14) | 94.13 (0.51) |
| 50 μg N | -3.48 (0.12) | 47.02 (0.49) | 49.14 (0.49) | 95.69 (0.88) |
| 75 μg N | -3.56 (0.05) | 73.24 (0.61) | 75.44 (0.64) | 97.08 (0.33) |
| 100 μg N | -3.76 (0.09) | 97.09 (0.45) | 100.60 (0.41) | 96.51 (0.27) |
| 150 μg N | -3.98 (0.03) | 147.17 (0.64) | 150.15 (0.64) | 98.01 (0.55) |

Supplement 2: Average atom % ^15^N and standard errors (N = 4) of N_2_O derived from a common NO_2_^–^ standard at 75 and 99 atom % ^15^N as produced using three NO_2_^–^ reducing bacterial cultures. *S. nitritireducens* is only capable of reducing NO_2_^–^ to N_2_O, while *P. chlororaphis* and *P. aureofaciens* can reduce both NO_3_^–^ and NO_2_^–^ to N_2_O.

|  | 75 atom % ^15^N | S.E | 99 atom % ^15^N | S.E. |
| --- | --- | --- | --- | --- |
| *S. nitritireducens* | 71.7 | 0.3 | 97.6 | 0.2 |
| *P. chlororaphis* | 45.5 | 3.2 | 58.3 | 1.9 |
| *P. aureofaciens* | 1.6 | 0.1 | 7.9 | 0.4 |
